# Vitamin D and cognition: Demographic disparities in memory recall and word intrusion in a multiethnic cohort

**DOI:** 10.1177/13872877251344604

**Published:** 2025-06-16

**Authors:** Juan C Lopez-Alvarenga, Isabel Omaña-Guzmán, Oscar Rosas-Carrasco, Jose E Cavazos, Michael C Mahaney, Gladys E Maestre

**Affiliations:** 1 Division of Population Health & Biostatistics, School of Medicine, University of Texas Rio Grande Valley, Edinburg, TX, USA; 2 Universidad Mexico Americana del Norte, Reynosa, Tamaulipas, Mexico; 3 Pediatric Obesity Clinic and Wellness Unit, Hospital General de México “Dr. Eduardo Liceaga”, Mexico City, Mexico; 4 Geriatric Assessment Center, Health Department, Iberoamerican University, Mexico City, Mexico; 5 Glenn Biggs Institute for Alzheimer's and Neurodegenerative Diseases, University of Texas Health Science Center at San Antonio, San Antonio, TX, USA; 6 Department of Neurology, University of Texas Health Science Center at San Antonio, San Antonio, TX, USA; 7 Department of Human Genetics, South Texas Diabetes and Obesity Institute, The University of Texas Rio Grande Valley School of Medicine, Brownsville, TX, USA; 8 Institute of Neurosciences, University of Texas Rio Grande Valley School of Medicine, Brownsville, TX, USA

**Keywords:** Alzheimer's disease, cognitive performance, ethnic groups, memory, minority groups, regression analysis, vitamin D, word intrusion

## Abstract

**Background:**

Vitamin D3 is essential for calcium metabolism and exerts pleiotropic effects, including neuroprotective activities in cognition. Its insufficiency has been linked to dementia, Alzheimer's disease, and cognitive impairments. The association between vitamin D3 and particular cognitive functions, including memory recall and word intrusion, remains imprecise, particularly among diverse ethnic and socioeconomic groups.

**Objective:**

To examine the relationship between vitamin D3 levels with memory recall and word intrusion in individuals aged 60 and above, emphasizing demographic differences.

**Methods:**

Data was collected from 2759 individuals in the NHANES 2011–2014 surveys. Cognitive performance was evaluated with the CERAD Word Learning, Animal Fluency, and Digit Symbol Substitution assessments. Factor analysis was employed to identify two cognitive domains: F1 ‘Memory Recall’ and F2 ‘Word Intrusion’. Linear and quantile regression models, controlled for demographic variables, were performed to assess the association between vitamin D3 levels and cognitive domains. Bootstrap techniques were used for standard error estimation, and nonparametric regression was applied to identify non-linear correlations.

**Results:**

Vitamin D3 levels positively correlated in linear models and quantile regression with F1 ‘Memory Recall’ at diminished cognitive function levels. F2 was not associated with vitamin D3. Socioeconomic factors influenced these correlations, revealing inequalities among ethnic and income groups.

**Conclusions:**

Elevated vitamin D3 levels correlate with improved memory recall, especially in individuals with lower cognitive percentiles. These findings suggest the potential of vitamin D3 to alleviate cognitive decline, including Alzheimer's disease, highlighting the need for focused interventions, particularly in underrepresented demographic groups.

## Introduction

Aging is associated with neurobiological changes that affect cognitive function. Approximately 40% of adults over 65 experience some degree of memory loss.^1^ While some of these cognitive changes are part of the normal aging process, others are associated with neurological disorders.

Vitamin D3 has been known for regulating bone calcium metabolism for over a century. A decade before the discovery of sunlight's effect on skin by converting 7-dehydrocholesterol to vitamin D3, the use of cod oil to prevent rickets was described.^2^ The well-known two-step activation of calcidiol [25(OH)D] and then calcitriol [1,25(OH)D3] involves interactions with enzymes like CYP27A1 and CYP2R1, complexes, and enzyme expression in a variety of tissues, suggesting an autocrine and paracrine mechanism with genomic and non-genomic effects.^3^

Recent studies have shown that vitamin D3 has pleiotropic effects as a hormone and acts as a neuroactive steroid, promoting the growth and functioning of the central nervous system.^4^ Vitamin D3 receptor (VDR) has been found in numerous brain cells, including oligodendrocytes, astrocytes, microglia, and neurons. Vitamin D3 deficiency has been linked with dementia, cognitive disorders, and an increased risk of Alzheimer's disease.^5^

However, the specific relationship between vitamin D3 and certain cognitive processes, such as memory recall and word intrusion, remains poorly understood. Memory recall, primarily facilitated by the hippocampus, is essential for retrieving factual information and events.^6^ By contrast, word intrusion, associated with the prefrontal cortex, involves the identification of mistakes and the capacity to differentiate between genuine and false memories.^7^ Moreover, the anterior cingulate cortex (ACC) detects errors, task anticipation, attention, and motivation. It also contributes significantly to recognizing inconsistencies, such as identifying words that were not initially included in the original list of the tests.^8,9^ The differential effect of vitamin D3 on these cognitive areas is inconsistent across studies due to factors (e.g., study design, population, measurements, and publication bias) that add complexity and require more rigorous research. Meta-analyses of randomized controlled trials found that vitamin D3 supplementation had a small but significant positive effect on global cognition in adults.^10,11^ Other studies failed to find significant cognitive benefits from vitamin D3 supplementation, suggesting no causal association between vitamin D3 and cognitive function.^12,13^

These relationships may be influenced by factors like sunlight exposure, dietary intake, and supplement use, which could vary across different ethnic and socioeconomic groups. We hypothesize that higher vitamin D3 levels are associated with higher memory scores and fewer word intrusion errors, with demographic variables potentially moderating these effects.

Despite the established roles of vitamin D3 in brain function and cognitive health, limited research has specifically examined how vitamin D3 influences distinct cognitive processes like memory recall and word intrusion. Thus, this study aimed to investigate whether serum vitamin D3 levels, ethnicity, and socioeconomic status were associated with variations in memory-related cognitive functions in older adults, using data from the 2011 and 2014 National Health and Nutrition Examination Survey (NHANES).

## Methods

NHANES was a complex, multistage probabilistic sample of civilian, noninstitutionalized residents of the 50 states and D.C. The 2011–14 sample design included an oversample of Asian Americans, among other races, and a description of Hispanic origin. The questionnaires were completed at home by trained interviewers using a Computer-Assisted Personal Interviewing (CAPI) system. The respondent selected the language of the interview (English or Spanish) or requested an interpreter be used. We collected data from 2759 individuals from the NHANES 2011 and 2014 surveys aged 60 and over.

### Cognitive tests

The Consortium to Establish a Registry for Alzheimer's Disease (CERAD) developed and standardized test batteries to assess cognitive changes associated with Alzheimer's disease.^14^ As a part of the neuropsychological battery, the CERAD Word Learning (CERAD-WL)^14,15^ assesses the ability to learn and remember new verbal information. It involves a list of 10 words, read aloud by the examiner, and the participant should recall as many words as possible. The number of intrusion words in each trial was recorded. After some learning trials, a delayed Word Learning Recall (CERAD-WLR) test is conducted to assess the retention of the words after a set period.

The Animal Fluency test^16^ evaluates verbal production, semantic memory, and language impairment. In this test, participants are asked to name as many animals as possible within one minute. The score is based on the total number of distinct animals named. In the NHANES, participants were given a practice task of naming three articles of clothing before the test. If they were unable to complete this task adequately, they did not proceed to the Animal Fluency Test.

The Digit Symbol Substitution Test (DSST),^17^ measures processing speed, attention, and working memory. It involves pairing symbols with numbers based on a key, and participants must match as many pairs as possible within a limited time. This test was part of the Wechsler Adult Intelligence Scale (WAIS III).

These cognitive tests provide valuable insights into learning, memory, language production, processing speed, and working memory; however, they often assess overlapping cognitive domains. By conducting multiple tests, researchers gather a comprehensive view of cognitive functioning that mitigates the limitations of any single measure. We used principal component analysis to combine the obtained scores to extract critical components (see Statistical Methods for details).

### Vitamin D

Serum concentrations of vitamin D3 (25-Hydroxyvitamin D [25(OH)D]) were measured using ultra-high performance liquid chromatography coupled with tandem mass spectrometry (UHPLC-MS/MS).^18^

### Statistical methods

Descriptive statistics of demographic variables with mean (SD) or frequency (percentages) according to the measurement were performed. Ethnic groups were classified as nominal variables, while household income was categorized as ordinal.

To assess the association of vitamin D3 levels, ethnicity, and socioeconomic status with cognitive tests we performed the following analyses:

1. Factor analysis. A principal component analysis (PCA) was conducted to determine whether the scores from the Word List Learning, Word List Recall, Animal Fluency, and Digit Symbol tests clustered into factors, to reduce data dimensionality. A varimax axis rotation was performed. To evaluate the suitability of the data for PCA, the Kaiser-Meyer-Olkin (KMO) test was conducted. The standard errors for the skewness and kurtosis of the identified factors were assessed using bootstrap methods with 10,000 repetitions. To optimize and consolidate this information, we gathered the obtained scores (i.e., Principal Components, which is a no-supervised analysis) to distill complex data into critical components, enhancing interpretability and enabling robustness. Data from multiple sources should support the cognition assessment so that the test results can be used assertively. The obtained factors were interpreted in the context of other variables like sex, ethnicity, and socioeconomic strata, giving a context of known background.^19^
2. Linear regression models. Adjusted linear regression models were performed to evaluate the association between vitamin D3 concentrations and the identified factors in the PCA. The models were adjusted by age, sex, ethnicity, household income, education, and body mass index (BMI).
3. A sensitivity analysis was conducted, utilizing each cognitive test (CERAD-WL Trials 1, 2, and 3; CERAD-WLR; Intrusion words in CERAD- WL and WLR; Animal Fluency Test; and DSST) as the dependent variable and vitamin D3 concentration as the independent variable in linear regression models adjusted by age, sex, ethnicity, household income, education, and BMI. The models were tested as stepwise backward regression, starting with all relevant predictors in the model. We used a threshold of p < 0.10 to be maintained in the model to ensure any relevant predictors remained.
4. Quantile regression models. These models were conducted to manage non-normality and skewed distributions of the identified factors in the factor analysis, employing Bootstrap with 1000 repetitions for standard error estimation. The models were adjusted by age, sex, ethnicity, household income per year, education, and BMI.
5. Additionally, we performed a quantile regression using least squares estimates of the conditional median and percentiles 25 and 75 of the response variable to obtain a more detailed description of the relations by quantiles of cognition scores. Models were adjusted by age, sex, ethnicity, household income, and BMI. The adjusted (predicted) values were evaluated with LOWESS (Locally Weighted Scatterplot Smoothing)^20^ and 95% confidence intervals to reveal the non-linear relationship. LOWESS is a nonparametric regression technique with no assumption of a specific functional form. We used a kernel Epanechnikov in the smoothing process, where observations closer to the target point receive more weight. With a bandwidth 8 to control the size of the neighborhood around each target point. Using LOWESS^20^ helps justify the presence of a relationship even when p-values are not significant for the middle and higher percentiles. It visually confirms the underlying pattern, highlighting a consistent positive association that traditional p-value thresholds might overlook.

All analyses were performed with Stata version 18.5 (StataCorp, College Station, TX).

### Ethics

NHANES 2011–14 is a large survey open-access dataset. The protocol for the surveys was approved by the NCHS Research Ethics Review Board.^21^ Our team analyzed the data without the involvement of interviewed human subjects.

## Results

We included 2759 individuals with a mean age of 69.4 (SD 6.8) years and a BMI of 29.1 (SD 6.4) kg/m^2^. Female participants were 51% (n = 1412). The included ethnic groups were Mexican Americans (9%), Other Hispanics (10%), Non-Hispanic Blacks (23%), Non-Hispanic Whites (49%), and Other ethnicity (10%). Household earnings less than $25k a year were 37%, and >$75k in 22% (Table 1).

**Table 1.** Demographic characteristics of the sample.

Variables	Frequency (percentage)
Sex (F/M)	1412 (51%) / 1347 (49%)
Age	
- 60 to <65 y	872 (32%)
- 65 to <70 y	632 (23%)
- 70 to <75 y	519 (19%)
- 75 and more y	736 (27%)
Ethnicity	
- Mexican Americans	245 (9%)
- Other Hispanics	281 (10%)
- Non-Hispanic Whites	1341 (49%)
- Non-Hispanic Blacks	630 (23%)
- Non-Hispanic Asians	226 (8%)
- Another race	36 (1%)
Attained Education	
- Less than 9th	307 (11%)
- 9 to 11^th^	384 (14%)
- High School/GED	647 (23%)
- Some college/AA	780 (28%)
- College and more	639 (23%)
Marital Status	
- Married	1529 (55%)
- Widowed	534 (19%)
- Divorced	389 (14%)
- Never married	159 (6%)
- Partner	74 (3%)
- Separated	72 (3%)
- Refused	2 (<1%)
Household income per year	
- Less than $9999	167 (6%)
- $10,000 to $24,999	819 (31%)
- $25,000 to $54,999	779 (30%)
- $55,000 to $74,999	287 (10%)
- $75,000 and more	585 (22%)

The denominators can vary according to the available data.

### Factor analysis

The loadings of factor analysis after varimax rotation showed two main factors explained 58% of the variance. Factor 1 included the scores of CERAD-WL and CERAD-WLR trials, Animal Fluency test and DSST, thus was identified as F1 ‘Memory Recall’. Factor 2 was identified as F2 ‘Word Intrusion’ since included the number of word intrusion in CERAD-WL trials. Table 2 presents varimax rotated loadings ≥0.5.

**Table 2.** Factor loadings with varimax rotation.

Variable	Factor 1	Factor 2	Uniqueness
Score CERAD-WL trial 1	0.77		0.39
Score CERAD-WL trial 2	0.84		0.29
Score CERAD-WL trial 3	0.83		0.31
Score CERAD-WLR	0.82		0.31
Intrusion word 1		0.7	0.5
Intrusion word 2		0.8	0.35
Intrusion word 3		0.78	0.38
Intrusion word recall		0.72	0.47
Score Animal Fluency	0.61		0.63
Score DSST	0.68		0.54

The table shows those loadings ≥0.5. CERAD-WL: Consortium to Establish a Registry for Alzheimer's Disease - Word Learning; CERAD-WLR: Word List Recall; DSST; Score Digital Symbol.

### Linear regression models

The adjusted linear regression showed a positive association between vitamin D3 concentrations and F1 ‘Memory Recall’ [β= 0.002 (95%CI 0.001, 0.003), p < 0.001] (Table 3). Meanwhile, F2 ‘Word Intrusion’ had an inverse marginal effect [β = −0.001 (95%CI: −0.002, 0.0001) p = 0.074]. The sensitivity analysis (Table 3) shows stability in the vitamin D3 effect on each studied cognitive test. Age was negatively associated with F1 ‘Memory Recall’ [β = −0.059] and with performance on each cognitive test. Female sex showed a positive association with F1 ‘Memory Recall’ [β = 0.314] and with cognitive test scores. Compared to Mexican Americans, Non-Hispanic White individuals scored higher on F1 ‘Memory Recall’ [β = 0.557] and on all cognitive tests. Similarly, Non-Hispanic Black and individuals of Other ethnicities demonstrated a positive association with F1 ‘Memory Recall’ [β = 0.116 and β = 0.301, respectively] and with all cognitive tests, except for the Animal Fluency test. Education level and BMI did not correlate significantly with F1 ‘Memory Recall’ or cognitive test performance.

**Table 3.** Sensitivity analysis: linear regression models for individual cognitive test scores and the composite F1 Memory Recall Factor.

Variable	CERAD-WL Trial 1	CERAD-WL Trial 2	CERAD-WL Trial 3	CERAD-WLR	Animal Fluency test	DSST	F1'Memory Recall’
	β	β	β	β	β	β	β
Age	−0.069***	−0.078***	−0.071***	−0.113***	−0.251***	−1.070***	−0.059***
Sex							
Female	0.479***	0.585***	0.515***	0.706***	−0.435**	4.298***	0.314***
Mexican Americans	Reference	Reference	Reference	Reference	Reference	Reference	Reference
Non-Hispanic white	0.780***	0.591***	0.620***	0.675***	2.224***	14.502***	0.557***
Non-Hispanic Black	0.496***	0.219*	0.250**	- - -	−1.384***	- - -	0.116*
Other ethnicity	0.377**	0.254*	0.437***	0.972***	−1.275***	10.753***	0.301***
Vitamin D (nmol/L)	0.002**	0.003***	0.003**	0.004**	0.010***	0.045***	0.002***
Body mass index (kg/m^2^)				0.015**			
Constant	8.607***	11.311***	11.596***	12.334***	33.077***	107.388***	3.524***
R^2^	0.1	0.111	0.089	0.14	0.139	0.288	0.199

The adjusted linear regression with stepwise backward departing for a model adjusted by age, sex, BMI, vitamin D concentration, and ethnicity as dummy variables. The reference group for ethnicity was Mexican American; the other Hispanic group was not different, nor were non-Hispanic blacks for some tests (CERAD-WLR and DSST), and, therefore, were dropped from the models. The BMI was removed from the model. Linear regression coefficients for covariates and scores for individual tests scores and F1 ‘Memory Recall’ in z-values. The coefficient of determination (R^2^) is at the bottom line. Factor 1 Memory Recall summarizes the six tests efficiently. DSST: Score Digital Symbol. *p < 0.1; **p < 0.05; ***p < 0.001.

### Quantile regression

The analysis of F1 ‘Memory Recall’ showed independent factors were age, sex, vitamin D3, BMI and ethnic groups (Adjusted R^2 = 0.20, p,0.001 with a root MSE = 0.089) (Table 4). The percentile 25 showed an independent effect from vitamin D, age, sex, ethnic group, and household income. Age was negatively associated [β = −0.0557]. Higher vitamin D3 concentrations and female sex were associated with higher cognitive performance [β= 0.0021 and β= 0.3712, respectively]. Compared with Mexican Americans, non-Hispanic white individuals and individuals of other Ethnicities had higher cognitive performance [β = 0.484 and β = 0.335, respectively]. Higher yearly household income was associated with higher cognitive scores.

**Table 4.** Quantile regression of F1 ‘Memory Recall’ and F2 “Word Intrusion (z-values) showing regression coefficients for vitamin D3 (nmol/L) adjusted by ethnic group, household income, and other demographic variables.

F1 Memory Recall	Q25	Q50	Q75
	β (95%CI)	β (95%CI)	β (95%CI)
Age	−0.056 (−0.064, −0.048)***	−0.057 (−0.063, −0.051)***	−0.0494 (−0.057, −0.042)***
Female	0.371 (0.267, 0.474)***	0.393 (0.308, 0.4778)***	0.417 (0.331, 0.503)***
BMI (kg/m^2^)	0.007 (−0.0004, 0.015)*	−0.0003 (−0.008, 0.007)	0.003 (−0.004, 0.010)
Vitamin D3 (nmol/L)	0.0021 (0.0003, 0.0039)**	0.001 (−0.0004, 0.0025)	0.0002 (−0.0012, 0.0015)
Mexican American	- Reference -		
Other Hispanic	−0.089 (−0.364, 0.186)	−0.159 (−0.329, 0.011)*	−0.260 (−0.479, −0.041)**
Non-Hispanic white	0.484 (0.246, 0.722)***	0.500 (0.362, 0.639)**	0.410 (0.215, 0.606)***
Non-Hispanic Black	0.066 (−0.185, 0.317)	0.129 (−0.015, 0.272)*	0.039 (−0.158, 0.237)
Other Ethnicity	0.335 (0.053, 0.618)*	0.304 (0.114, 0.495)**	0.234 (0.011, 0.457)**
Household income <$10k	- Reference -		
$10k to < $25k	0.317 (0.124, 0.511)***	0.268 (0.060, 0.476)**	0.230 (0.058, 0.401)***
$25k to <$55k	0.522 (0.324, 0.719)***	0.472 (0.259, 0.684)***	0.391 (0.215, 0.567)***
$55k to <$75K	0.776 (0.547, 1.005)***	0.6256 (0.406, 0.845)***	0.520 (0.314, 0.726)***
>$75k	0.834 (0.635, 1.033)***	0.732 (0.516, 0.948)***	0.656 (0.475, 0.837)***
Constant	2.036 (1.317, 2.755)***	3.025 (2.515, 3.535)***	3.154 (2.517, 3.791)***

F2 Intrusion	Q25	Q50	Q75
	β (95%CI)	β (95%CI)	β (95%CI)
Age	−0.006 (−0.007, −0.005)***	−0.004 (−0.005, −0.003)***	0.010 (0.001, 0.019)**
Female	0.0382 (0.0223, 0.0541)***	0.0349 (0.0167, 0.0532)***	0.012 (−0.0984, 0.1224)
BMI	−0.001 (−0.0017, −0.006)	−0.0001 (−0.0029, 0.0005)	−0.0045 (−0.0139, 0.0048)
Vitamin D3 (nmol/L)	0.0001 (−0.0001, 0.0004)	−0.0001 (−0.0004, 0.0002)	−0.0004 (−0.0022, 0.0013)
Mexican American	- Reference -		
Other Hispanic	−0.011 (−0.043, 0.020)	0.006 (−0.08, 0.095)	0.413 (0.108, 0.718)***
Non-Hispanic white	0.051 (0.027, 0.076)***	0.041 (0.001, 0.082)**	−0.045 (−0.221, 0.132)
Non-Hispanic Black	0.026 (−0.002, 0.053)*	0.058 (0.003, 0.112)**	0.485 (0.2485, 0.711)***
Other ethnicity	0.004 (−0.025, 0.033)	−0.015 (−0.060, 0.031)	−0.152 (−0.375, 0.072)
Household income <$10k	- Reference -		
$10k to < $25k	0.038 (0.003, 0.073)**	−0.005 (−0.082, 0.071)	0.267 (−0.025, 0.559)*
$25k to <$55k	0.057 (0.023, 0.091)***	0.001 (−0.073, 0.074)	0.015 (−0.257, 0.287)
$55k to <$75K	0.078 (0.041, 0.116)***	0.032 (−0.044, 0.107)	0.072 (−0.245, 0.388)
>$75k	0.087 (0.050, 0.124)***	0.032 (−0.042, 0.106)	0.043 (−0.231, 0.316)
Constant	−0.207 (−0.304, −0.110)***	−0.133 (−0.274, 0.008)*	−0.667 (−1.389, 0.047)*

The described coefficients are from quantile regression on 25, 50, and 75 percentiles. The standard errors were calculated with 1000 bootstrap repeats. The effects by quartile showed different associations. The F1 Memory Recall showed that vitamin D3 has the maximum negative effect in the lower quartile.

*p < 0.1, **p < 0.05, ***p < 0.001.

While differences in cognitive performance were found between ethnic and household income groups, vitamin D3 was associated with higher scores regardless of ethnicity and household income.

In the analysis of the percentile 75 of F1 ‘Memory Recall’, vitamin D3 was not significantly associated with cognitive performance. The predictors in this group were age ([β = −0.049], sex [β = 0.417 for females], ethnicity, and yearly household income. As in the percentile 25, non-Hispanic white individuals [= 0.410] and individuals classified as Other Ethnicity [= 0.457] showed higher cognitive scores than Mexican Americans. In contrast, people identified as Other Hispanic had lower cognitive performance [β = −0.260]

For F2 ‘Word Intrusion’, no significant association was observed with vitamin D3 at any percentile. However, age, sex, ethnicity, and household income were most notably associated with cognitive outcomes in the percentile 25 (Table 4).

### Smoothing

LOWESS does not assume a specific functional form and demonstrated an evident positive trend across the spectrum of vitamin D3 levels, reinforcing the idea that the relationship may be more complex than a simple linear association captured by traditional regression methods.

**Figure 1.**
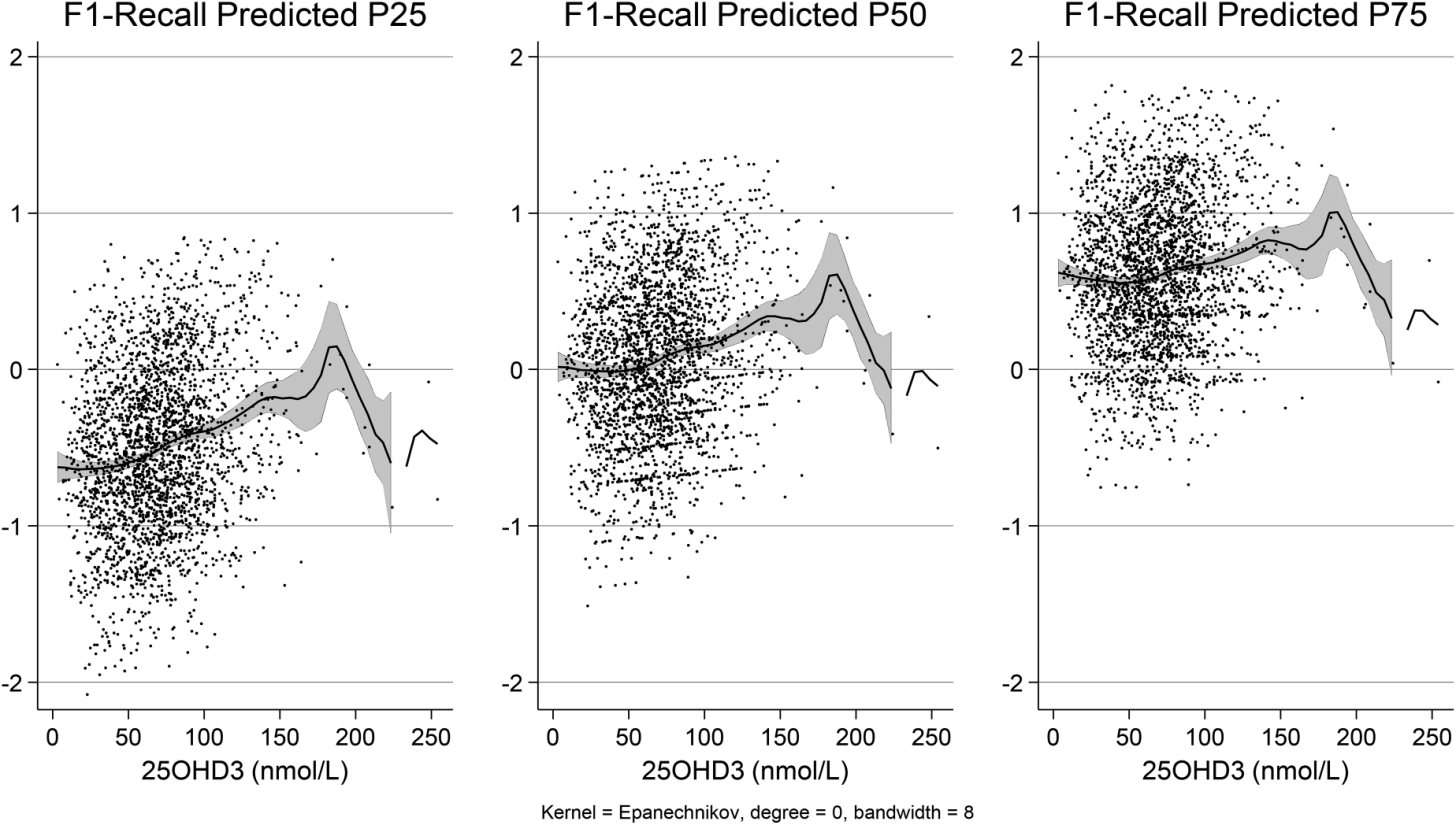
Scores of the F1 ‘Memory Recall’ on vitamin D3 serum concentration. The figure describes three distributions based on 25, 50, and 75 percentiles of the scores. The LOWESS (Locally Weighted Scatterplot Smoothing) visualized a non-linear relationship, reinforcing the concept of a more complex function than a simple linear association. Those in the first quartile (percentile 25) have a greater slope than others. The quantile regression was adjusted by age, sex, BMI, race, education attained, and economic strata.

**Figure 2.**
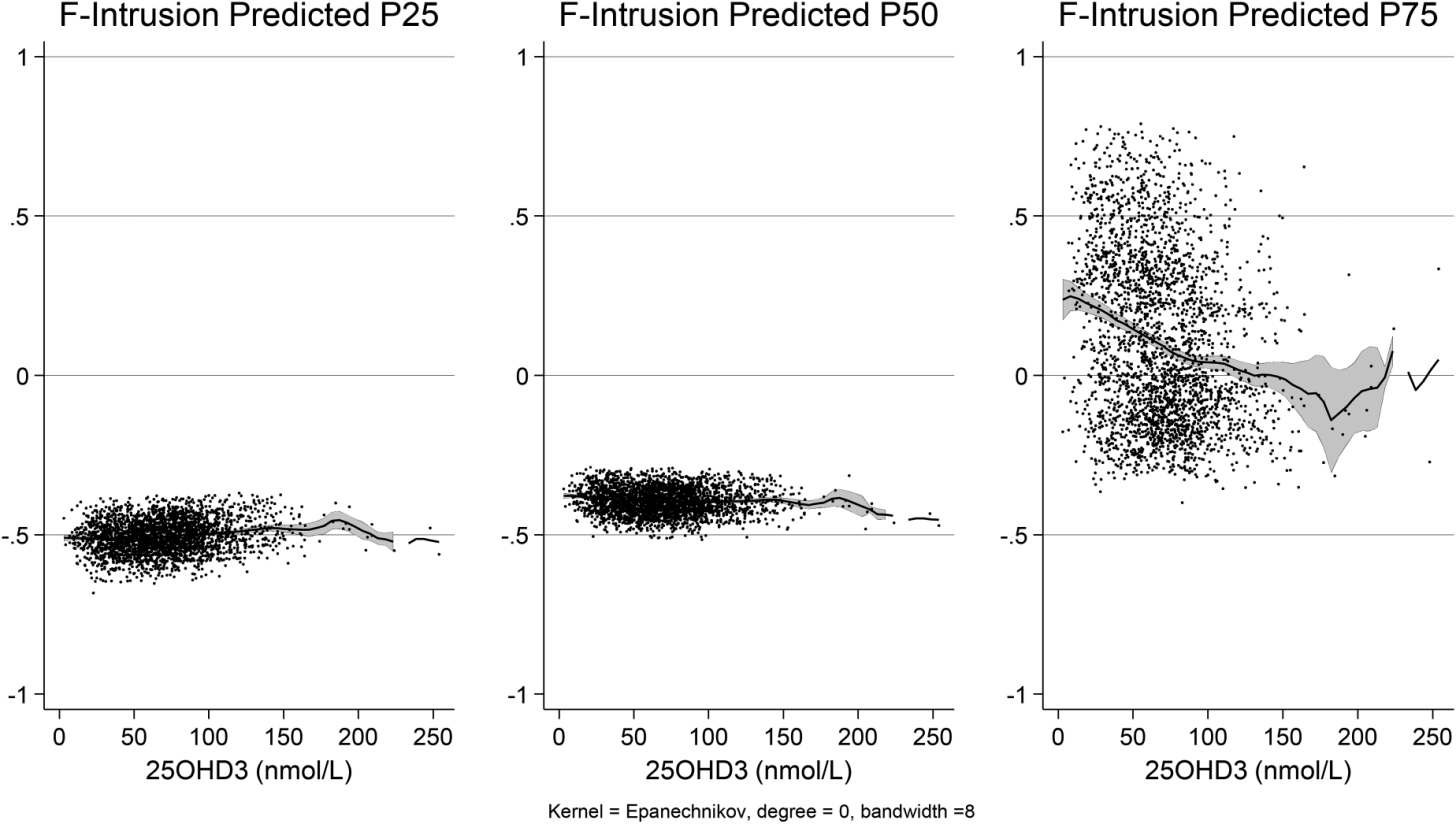
Scores of the F2 ‘Word Intrusion’. The figure describes three distributions based on 25, 50, and 75 percentiles of the scores. The LOWESS (Locally Weighted Scatterplot Smoothing) visualized a non-linear relationship. Those in the third quartile (percentile 75) have a greater negative slope compared to other quartiles, indicating an increase in intrusion score with lower levels of vitamin D3. The quantile regression was adjusted by age, sex, BMI, race, education attained, and economic strata.

This smoothing approach with 95% confidence intervals (Figures 1 and 2) supports the concept that vitamin D3's effect on cognition might be nonlinear, and the impact could vary in strength depending on the concentration range. The more pronounced relationship at the lower percentiles and a gradual increase observed in the middle and upper ranges suggest that cognitive benefits may accumulate differently across vitamin D3 levels.

## Discussion

This study is among the first to explore the relationship between vitamin D3 concentrations and cognitive performance, specifically memory recall and word intrusion, across different ethnic and socioeconomic groups. Our analysis demonstrated that vitamin D3 levels, especially in the 25th percentile, were significantly associated with memory recall, even after adjusting for demographic factors such as sex, age, BMI, education, ethnicity, and household income in older adults. While the regression coefficients for the 50th and 75th percentiles were not statistically significant, the overall trend across all percentiles suggested a positive relationship between vitamin D3 levels and memory recall.

Our research highlights the significance of socio-demographic variables in cognitive performance and indicates that enhancing vitamin D3 levels may mitigate cognitive decline. These findings provide insights into potential preventive efforts for dementia and Alzheimer's disease, particularly in diverse and socioeconomically disadvantaged populations. The quantile regression results suggest that socioeconomic status, as measured by household income, is positively associated with memory recall across all income levels, with individuals in higher income brackets showing significantly better cognitive performance. This finding aligns with evidence that socioeconomic advantages may provide greater access to healthcare, nutrition, and resources that support cognitive health in aging. Besides, the complexity increases when considering biological factors demonstrated in previous studies that vitamin D is involved in several neurological functions, especially in the hippocampus, prefrontal cortex, and anterior cingulate cortex (ACC), critical areas for memory retrieval and verbal intrusion.^22^

Vitamin D3 plays a role in cell differentiation, neurotrophins and neurotransmitter synthesis, intracellular calcium regulation, and antioxidant activity, influencing brain and cognitive functions.^23^ Although the relationship between vitamin D3 deficiency and cognitive decline is still not well understood, several pathophysiological mechanisms have been identified. One key mechanism involves impaired neurogenesis. Vitamin D3 deficiency disrupts remyelination, reducing cell proliferation, survival, and differentiation of oligodendrocytes and neurons.^24,25^ In 1-alpha hydroxylase knockout mice, which lack the ability to produce active forms of vitamin D3, neurogenesis is delayed in adulthood. It has been shown that a vitamin D-deficient diet in mice increases proliferation in the dentate gyrus of the hippocampus during early development but decreases cell survival in adulthood. Additionally, mice lacking vitamin D3 receptors exhibit reduced neuronal differentiation.^3,26^

Another mechanism that is affected by vitamin D3 deficiency is the synaptic plasticity, a crucial element for learning and memory processes. The deficiency of this vitamin disturbs the processes, adversely affecting synaptic plasticity,^23^ and is also associated with changes in the expression of genes related to plasticity, such as Drebrin (developmentally regulated brain protein) and neuromodulin.^27^ A study using aged rats demonstrated that vitamin D3 administration, combined with environmental enrichment (EE), augmented synaptic plasticity and enhanced learning and memory abilities.^28^ In a rat model of unpredictable chronic mild stress, another study found that vitamin D3 inhibited the increase of amyloid-beta (Aβ) and the reduction of brain-derived neurotrophic factor (BDNF) in the hippocampus, thereby enhancing learning and memory.^29^

Vitamin D3 supplementation has shown inconsistent results regarding its effectiveness in improving cognitive function. Studies in animal models have shown that vitamin D3 supplementation could prevent or improve neurodegenerative diseases by its anti-inflammatory and antioxidant capacity, regulating calcium homeostasis, and reducing and inhibiting the synthesis of pathogenic proteins, among other mechanisms.^30^ However, epidemiological studies have reported inconsistent findings on the cognitive benefits of vitamin D3 supplementation. Our research contributes to the growing body of evidence suggesting that vitamin D3 may improve cognitive performance, especially in individuals with low vitamin D3 levels or pre-existing cognitive deficits.^10,13,31^

Studies have shown that sociodemographic and lifestyle variables are associated with cognitive performance in adults.^32–34^ A previous study carried out in Brazil^33^ found an association between poor family structure including low income in childhood, food insecurity, with poorer performance in adulthood in word recall, and semantic and phonemic verbal fluency tests. We found cognitive differences between ethnic and household income groups. Both ethnicity and socioeconomic status are related with different lifestyle factors and social determinants of health.^35^ Similarly to our findings, other studies have found disparities in cognitive function among ethnic groups, with Non-Hispanic White individuals showing the highest performance.^36^ We did not find that ethnicity and household income modified the effect of vitamin D3; regardless of these variables, vitamin D3 was associated with higher cognitive performance.

The finding that females generally performed better on memory recall has been reported before,^37^ indicating that women may have a cognitive advantage in certain domains, which could influence their resilience to cognitive decline.^38^ However, it is also known that postmenopausal women may experience more rapid cognitive decline and have a higher prevalence of Alzheimer's disease than men,^39–41^ possibly due to hormonal changes and longevity.^42–44^

Our study had some limitations. The observational design restricts our ability to infer causality, and the variability in participants’ socioeconomic backgrounds introduces complexity in interpreting the associations with race and education. Additionally, use of broad cognitive factors (memory and intrusion) to capture overall cognitive profiles may dilute specific cognitive abilities. While combining tests enhances statistical power, it may obscure unique contributions from individual cognitive processes. A sensitivity analysis confirmed that our factors represented meaningful cognitive dimensions, but future studies should examine these constructs more closely.

The non-linear link between vitamin D3 and cognition was captured using non-parametric approaches like LOWESS, which showed that vitamin D3 had a greater effect at lower levels. This analysis strategy emphasizes the relevance of non-linear correlations, which standard regression approaches are likely to overlook.

Future studies should investigate how vitamin D3 impacts memory recall and word intrusion, especially across demographic groupings. RCTs and longitudinal studies could establish the efficacy of tailored vitamin D3 therapies. Neuroimaging could also reveal vitamin D's neuronal mechanisms of cognition, leading to potential treatments.

Efforts to promote successful cognitive aging have focused on shifting the trajectory of cognitive decline by enhancing the cognitive reserve.^45^ The cognitive reserve model suggests that symptoms of cognitive impairment appear only when the number of functioning neurons, or their connections, fall below a critical threshold.^46^ Sufficient levels of vitamin D3 may help maintain a healthy brain and delay the onset of cognitive symptoms. Future studies should explore the potential neuroprotective effect of vitamin D3 on various mechanisms related to cognitive aging.

In conclusion, this study demonstrates that age, sex, vitamin D3 levels, and ethnicity are significant predictors of memory recall. The varying relationships between socioeconomic status and cognitive performance underscore the need for targeted interventions to promote cognitive health in diverse populations. Addressing these gaps can help mitigate cognitive decline, including Alzheimer's disease, and improve cognitive outcomes across different demographic groups.
